# Conformational Effects on the Circular Dichroism of Human Carbonic Anhydrase II: A Multilevel Computational Study

**DOI:** 10.1371/journal.pone.0056874

**Published:** 2013-02-25

**Authors:** Tatyana G. Karabencheva-Christova, Uno Carlsson, Kia Balali-Mood, Gary W. Black, Christo Z. Christov

**Affiliations:** 1 Department of Applied Sciences, School of Health and Life Sciences, Northumbria University, Newcastle upon Tyne, United Kingdom; 2 IFM-Department of Chemistry, Linköping University, Linköping, Sweden; 3 Department of Biochemistry, Oxford University, Oxford, United Kingdom; University of South Florida College of Medicine, United States of America

## Abstract

Circular Dichroism (CD) spectroscopy is a powerful method for investigating conformational changes in proteins and therefore has numerous applications in structural and molecular biology. Here a computational investigation of the CD spectrum of the Human Carbonic Anhydrase II (HCAII), with main focus on the near-UV CD spectra of the wild-type enzyme and it seven tryptophan mutant forms, is presented and compared to experimental studies. Multilevel computational methods (Molecular Dynamics, Semiempirical Quantum Mechanics, Time-Dependent Density Functional Theory) were applied in order to gain insight into the mechanisms of interaction between the aromatic chromophores within the protein environment and understand how the conformational flexibility of the protein influences these mechanisms. The analysis suggests that combining CD semi empirical calculations, crystal structures and molecular dynamics (MD) could help in achieving a better agreement between the computed and experimental protein spectra and provide some unique insight into the dynamic nature of the mechanisms of chromophore interactions.

## Introduction

*Circular Dichroism* (CD) spectroscopy is a highly utilized method for the investigation of protein structure [1]. In the near-UV region (240–320 nm) the method is used to identify delicate structural changes related to the orientation of the protein aromatic and disulfide amino-acids side chains, which might be a result of their interactions with ligands and mutations. In the far-UV region (190–240 nm) the method is used to characterize changes in the secondary structure of proteins. The aromatic side chain chromophores, such as tryptophans and tyrosines, have the greatest contribution to the near-UV region of the CD spectra but can also contribute to the far-UV intensities [2], [3].

The fundamental molecular unit of CD is the *Rotational Strength* which is defined as the imaginary part of the scalar product between the electric and magnetic transition dipole moments [4]. Most protein chromophores, however, including the aromatic ones, are not intrinsically chiral, contain elements of mirror symmetry and therefore have zero rotational strengths and no CD spectrum. Within the protein environment these chromophores become chirally perturbed and generate rotational strengths by three mechanisms [5] namely: i) *the one-electron mechanism* (intra-chromophore mixing) - mixing of electrically and magnetically allowed transition moments within the same chromophore; ii) *the µ- µ mechanism* - coupling between electrically allowed transitions in two separate chromophores; and iii) *the µ-m mechanism -* coupling between electrically and magnetically allowed transitions in two separate chromophores. The last two are also known as *coupled-oscillator type* (inter-chromophore mixings) mechanisms to reflect that the interactions are between two different chromophores.

Despite the huge amount of data available on protein structures and the increased implementation of CD, the contributions of the aromatic side chains have not yet been entirely revealed. Such knowledge would explains effects of mutations, alterations in the local protein structure, characterization of reaction intermediates, ligand interactions and monitoring of the folding process, thus providing a better understanding of protein structure-function relationships [2], [6], [7].

Proteins such as the Human Carbonic Anhydrase (HCAII) are characterized by remarkably complex contributions of the aromatic chromophores (mainly from the seven tryptophans and eight tyrosines) to the CD spectra. A comprehensive experimental investigation of the wild-type enzyme and seven tryptophan mutant forms of the enzyme revealed that the tryptophan chromophores not only determine the near-UV CD spectral features of the protein but also contribute sensitively to the far-UV region [8]. In addition the CD spectrum of the wild type enzyme was calculated using the matrix method [9], with *ab initio* monopoles. Calculations of the CD spectra of the tryptophan mutants were done by the matrix method using semi-empirical monopoles [10] and in the case for W192F *ab initio* monopoles were used [9]. All calculations are based on single crystal structures. The experimental CD spectrum of HCAII in the near-UV region is considered as complex, and indicative of complicated aromatic chromophore interactions [8]. The recent development of computational chemistry methods and high performance computing provides advanced opportunities for analyzing such complex protein spectral properties which are potentially insightful for better understanding of protein structure-function relationships.

**Figure 1:**
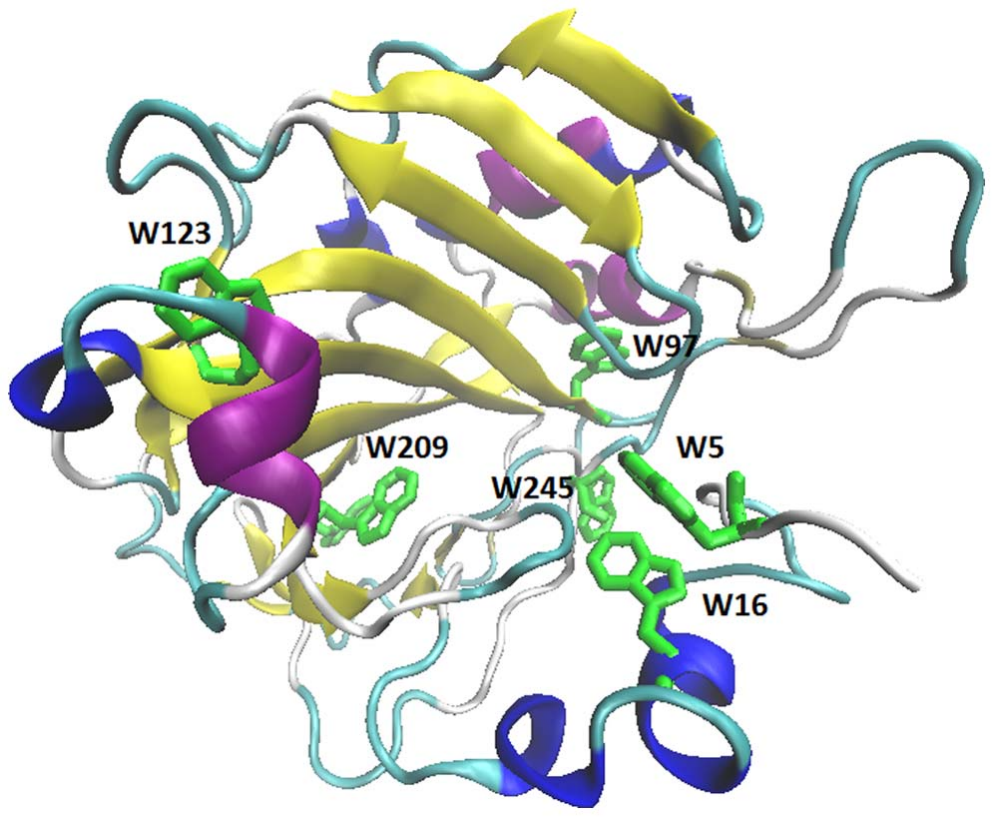
Structure of HCAII. The tryptophan chromophores are shown *in blue licorice.*

Carbonic anhydrase (EC 4.2.1.1) is a zinc-containing metalloenzyme that catalyzes the reversible conversion of carbon dioxide to a bicarbonate anion and a proton [11]. The enzyme form studied here, the Human Carbonic Anhydrase II (HCAII), is located in erythrocytes and is one of the most active enzymes known to date. It consists of one polypeptide chain organized in a single domain protein without any disulfide bonds. The structure is primarily dominated by a β-sheet which spans along the entire molecule and has a small α-helical content (Figure 1). Relative to the average protein in humans, Trp is about twice as abundant in HCAII (2.7% vs 1.4%), whereas the abundance of the Tyr in HCAII is comparable to that in the average protein (3.1% vs 3.2%). [12]. It has also been shown experimentally that these chromophores and their interactions have a strong impact on the near-UV and far-UV CD [8]. Tryptophans W97, W123, W192, W209 and W245 are positioned in a β-sheet with tryptophan; W97 being deeply buried. In addition tryptophans W5, W16 and W97 are located in aromatic clusters, which might influence the coupling interactions between them that would reflect in the resulting CD spectrum.

Nevertheless, recent studies do not facilitate a better understanding of the underlying mechanisms of interaction between the aromatic chromophores which generate the CD spectra. In addition, due to the protein conformational flexibility these aromatic interactions would potentially have some dynamic nature which is important to explore. Providing such insight could be an excellent opportunity to demonstrate the synergy effect from integrated application of multilevel computational methods in correlation with the available structural and spectroscopic data.

This paper presents a comprehensive multilevel computational study of the CD properties of HCAII in correlation with the experimental CD spectra, which is performed with the following objectives:

- understanding the mechanisms of generation of the near-UV CD spectrum by means of interactions between the transitions of the aromatic chromophores;
- evaluating the impact of the protein conformational flexibility on the quality of the calculated spectra;
- exploring the sensitivity of chromophore interactions identified in the near-UV to the effect of the protein conformational dynamics;
- computing the effects of tryptophan mutations on the CD spectra in correlation with the experimental ones;
- evaluating the applicability of restricted structural model including only the tryptophan and tyrosine chromophores at both semiempirical level (using the matrix method) and Time-Dependent Density Functional Theory (TDDFT);

This study is focused mainly on the aromatic contributions (*Lb* and *La* transitions) in the near-UV CD. Indeed the higher energy aromatic transitions (*Bb* and *Ba*) might contribute sensitively to the far-UV [3], [10] where they mix with a huge number of peptide transitions. The analysis of the interactions would be therefore complicated and is not present here.

## Methods

Three levels of modelling methods were carried out in the study of HCAII CD spectral features: i) *Atomistic Molecular Dynamics* (MD) simulations [13], [14]; ii) *Approximate Quantum Mechanical* CD calculations using the Matrix Method [15] and iii) *Time Dependent Density Functional Theory* (TDDFT) calculations [16]. Tryptophan mutant structures were prepared by *in silico* mutagenesis from the crystal structure of the wild-type of HCAII taken from Protein Data Bank (Berman and others 2000) (PDB ID code 2cba) (Hakansson and others 1992), and structural snapshots of the wild-type protein and tryptophan mutant forms were taken from MD simulations.

The CD calculations with the matrix method were performed incorporating all peptides and side chain chromophores. The matrix method calculations were performed using the *Dichrocalc* web interface [17]. This method [15] in its origin-independent form [18] considers the protein as a system of *M* independent chromophoric groups. The wave function of the entire molecule is represented as a linear superposition of basis functions. Every basis function is a product of all monomer wave functions where only one group is in an excited state. This way the matrix method incorporates all mechanisms of generation of the rotational strengths (*μ-μ*, *μ-m* and the static field effect). The interactions between the chromophores are considered to be purely electrostatic and therefore the permanent and transition electron densities (represented by monopoles) are implemented from electronic structure calculations on model systems. Finally, the Hamiltonian matrix is diagonalized by unitary transformation in order to represent the excited states in the interacting system. More details about the matrix method can be found in [5], [19], [20]. The monopoles for the side chain chromophores (including the aromatic ones) are taken from *ab initio* calculations [21] and the monopoles for the peptide chromophores are taken from *ab intio* [22] and semi-empirical calculations [23].

TDDFT calculations were done with Gaussian09 code [24] and to the best of our knowledge represent one of the largest biomolecular TDDFT calculations. The system included only 3-methylindole parts from the side chains of the tryptophans and the phenol parts from the side chains for the tyrosines kept at their positions from the crystal structure with the rest of the system being deleted due to computational demands. The missing hydrogen atoms were added using GausView5 [25]. Continuum solvent model with a dielectric constant of 4 was used to approximately represent the protein environment. The B3LYP functional, with three basis sets (6-31G(d), 6-31G(d,p) and 6-31++G(d,p)), was used as it was previously demonstrated that this model provides reasonable results for tryptophan zipper proteins [26]. A comparison of the different basis sets is provided in Figure S1 in Supporting Information S1 and here we will focus only on B3LYP/6-31G(d) results of the wild-type and all seven tryotophan mutants.

The MD simulations were carried out with the GROMACS code (version 4.3.1) [27] and Gromos43b1 force field for 20 nanoseconds (ns) for the wild-type enzyme and all seven tryptophan mutants. The system was prepared from the crystal structure of HCAII using Gromacs utilities for system preparation. The hydrogen atoms were added and geometry was energy minimized according to the protonation states of the ionogenic groups. Consequently the entire system was minimized using the steepest descent algorithm. The system was solvated using rectangular SPC water box placed 10 Å from the edges of the protein, neutralized and the solvent was equilibrated for 50 picoseconds (ps). Production MD was run for 20 ns in NPT ensemble at 310K applying Berendsen thermostat [28]. The electrostatic interactions were treated by Particle Mesh Ewald method [29]. The quality of the simulations was monitored by RMSDs (Figure S2 in Supporting Information S1).

The structures of the seven mutants were prepared from the crystal structure of the wild- type enzyme using the *What if* web interface (http://swift.cmbi.ru.nl/servers/html/index.html) [30]. Consistent with the experimental CD studies of HCAII tryptophan mutants [8], the following structures: W5F, W16F, W97C, W123C, W192F, W209F and W245C were prepared. The received structures were additionally energy minimized to avoid local stretching interactions. All structures for MD simulations were prepared as in the case for the wild-type enzyme. The protein structure of HCAII was visualized using VMD [31]. The experimental CD spectra were taken from [8].

## Results and Discussion

### CD Spectrum of the Wild-type HCAII Based on the Crystal Structure

**Figure 2:**
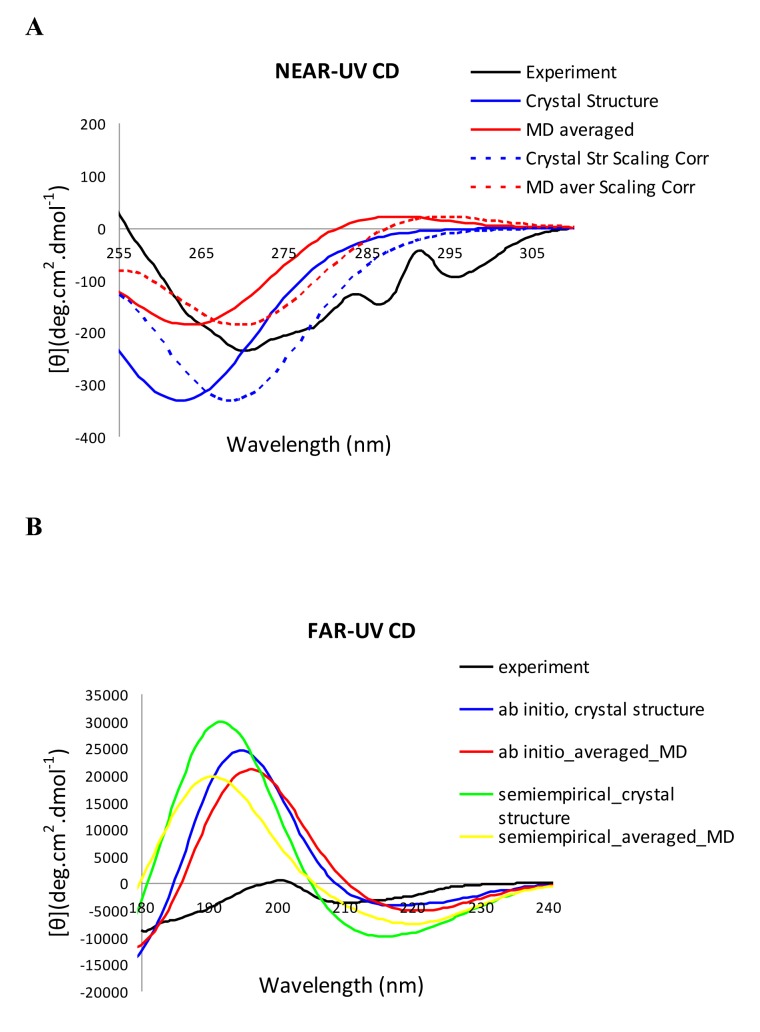
Calculated and experimental CD spectra of HCAII. **A.** Near-UV: the experiment (*black, continuous line*); calculated using single crystal structure (*blue, continuous line*); averaged calculated spectrum using MD snapshots (*red, continuous line*); calculations using single crystal structure after scaling correction - red shifting by 6 nm (*blue dotted line*); averaged calculated spectrum using MD snapshots after scaling correction - red shifting by 6 nm (*red dotted line*); **B.** Far-UV: the experiment (*in black*); calculated with *ab initio* peptide chromophores using the crystal structure (*in blue*); with semi-empirical peptide chromophores and the crystal structure (*in green*); with *ab initio* chromophores based on MD snapshots (*in red*); with semi-empirical chromophores based on MD snapshots (*in yellow*).

The near-UV CD spectrum of the wild-type enzyme calculated with the matrix method using the crystal structure in comparison to the experimental spectrum is presented in Figure 2A (the computed spectrum is shown in blue and the experimental spectrum is shown in black). The calculated spectrum is characterized by a spectral minimum (at 263 nm) and represents the correct spectral sign and overall shape, however, the magnitude at the spectral minimum is deeper than the experimental one (by 94 deg.cm^2^.dmol^−1^). The position of the minimum of the calculated spectrum is blue-shifted by 7 nm in respect to the experimental spectrum as in the calculations done by Hirst et al. performed with the same model and parameters [9]. The achieved level of agreement is reasonable for the semiempirical matrix method we apply, however applying potentially more accurate methods such as TDDFT on the system is not feasible at present. In the experimental spectrum, there are features above 280 nm due to the fine vibration structure, not reproduced in the calculated spectrum. At present, however, it is almost impossible to reproduce such features in extremely large systems as proteins, including huge number of chromophores. Even in much smaller molecules and applying more accurate methods might be hard to reproduce such features. The calculations confirm that the tryptophan chromophores generate the dominant part of the near-UV rotational strengths of the CD spectra and the tyrosines exhibit lower contributions (Figure S3 in Supporting Information S1).

The far-UV CD spectrum was calculated by means of two sets of monopoles for the peptide chromophore - semi-empirical ones by Woody [23] (Figure 2B, in green) and *ab initio* ones by Hirst [22] (Figure 2B, in blue). Whilst the experimental spectral magnitudes (Figure 2B, in black) are not well reproduced in either cases, the *ab initio* monopoles provide a slightly better representation in the far-UV CD (Figure 2B).

### Mechanistic Insight: Interactions between the Aromatic Chromophores

The qualitative reproduction of the near UV CD spectrum provides the opportunity to analyze the mechanisms by which the individual chromophores interact in order to generate rotational strengths (Table 1). The one electron type of interactions (intra-chromophore mixing) are generated by all tryptophan and most of the tyrosine chromophores. The most significant interaction energies exhibit the mixing between the *Lb* and *La* transitions of W123, and the mixing between *Lb* and *La* transitions of W209. Tryptophans also participate in a coupled oscillator type of interactions (mixing of transitions between different chromophores) with other tryptophan and tyrosine chromophores. For example, there are interactions between the transitions of W5 and W16 (spaced at 5.4 Å); W97 and W245 (8.0 Å); W192 and W209 (10.4 Å); W123 and Y128 (10.1 Å); W192 and Y191 (8.6 Å); and Y194 and W209 (3.9 Å). Nevertheless, it is clear that tryptophans participate in several coupling interactions: the one electron mixing type of interactions tend to exhibit higher interaction energies with at least one order of magnitude higher than the coupled oscillator type ones (Table 1). The results are in agreement with earlier studies on class A β-lactamases, which revealed that the one electron effect is the prefered mechanism by which tryptophans generate the strongest contributions to the near-UV CD spectra [20], [32], [33].

**Table 1:** Interactions between the aromatic chromophores in the near-UV CD of the wild type HCAII.

Res 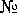 /Chromophore/Transition	Res 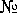 /Chromophore/Transition	Distance(Å)	Interaction Energy (cm^−1^)
5W-*Lb*	5W-*La*	0.0	389.20
5W-*Lb*	16W-*Lb*	5.4	−0.32
5W-*Lb*	16W-*La*	5.4	−39.77
5W-*La*	16W-*Lb*	5.4	−40.33
5W-*La*	16W-*La*	5.4	−67.04
16W-*Lb*	16W-*La*	0.0	400.77
97W-*Lb*	97W-*La*	0.0	306.39
97W-*La*	245W-*La*	8.0	7.80
123W-*Lb*	123W-*La*	0.0	993.97
192W-*Lb*	192W-*La*	0.0	14.74
209W-*Lb*	209W-*La*	0.0	460.83
245W-*Lb*	245W-*La*	0.0	−416.43
192W-*La*	209W-*La*	10.4	12.27
7Y-*Lb*	7Y-*La*	0.0	−120.69
40Y-*Lb*	40Y-*La*	0.0	−220.06
51Y-*Lb*	51Y-*La*	0.0	28.62
114Y-*Lb*	114Y-*La*	0.0	293.43
128Y-*Lb*	128Y-*La*	0.0	192.34
191Y-*Lb*	191Y-*La*	0.0	28.61
194Y-*Lb*	194Y-*La*	0.0	87.52
128Y-*La*	123W-*La*	10.1	−15.18
191Y-*La*	192W-*La*	8.6	−16.82
88Y-*La*	128Y-*La*	7.8	9.65
194Y-*La*	209W-*La*	3.9	−93.07

The first two columns contain residue numbers and transitions. The third column contains the distance between the residues. The last column contains the interaction energy.

### Influence of Conformational Flexibility on the Calculated CD Spectra of the Wild- Type HCAII

Proteins are characterized by intrinsic conformational flexibility which might influence their structural properties and functions [34], [35] and MD is one of the most widely utilized techniques for exploration of their conformational dynamics [36]. Since CD spectra are a consequence of the mutual orientation and distances of the protein chromophores within the protein structure, conformational flexibility would exercise an influence on the chiroptical properties of proteins, e.g. on the quality of the predicted CD spectra and the nature of the underlying mechanisms. To explore this important issue 20 ns MD simulations of the wild-type enzyme were performed and the CD spectra using 40 random structures (snapshots) along the MD trajectory were calculated. The averaged spectrum over the calculated MD snapshots provides almost a two-fold better agreement to the experimental one for the main near-UV spectral feature (the minimum at 270 nm in the experimental spectrum and 263 nm in the calculated one), in contrast to the calculated spectrum based on the X-ray crystal structure alone (Figure 2A, in red). In order to facilitate the comparison, we presented also scaled computed spectra which were received through red shifting of the original ones by 6 nm (presented in Figure 2A with dashed blue and dashed red lines, respectively for the crystal structure and MD-averaged scaled spectra). Up to 267 nm (275 nm for the scaled spectra) the MD averaged calculations provide better agreement to the experimental one, and above this wavelength the calculations based on the crystal structure show closer magnitudes to the experiment. Above 280 nm (287 nm for the scaling corrected spectra) the MD-based spectrum shows slightly positive sign (in contrast to the experiment and the calculations based on the crystal structure only). This could be due to interactions in non-favorable protein conformations. Its intensity, however, is relatively small and would not diminish the better agreement achieved for the main spectral feature. In the far-UV region, the averaged spectra calculated over the MD snapshots provide some improvement to the predictions of the CD spectral magnitudes as well, however the results are still far from being in a good agreement with the experimental data (Fig. 2B, with semi-empirical monopoles *in yellow*, and with *ab initio* ones *in red*).

### Mechanistic Effects of the Conformational Changes

Combining CD calculations and MD enables exploration of the influence of the protein conformational flexibility on the mechanisms of generation of rotational strengths and chromophore interactions, thus facilitating a deeper insight into the protein structure-spectra relationship. Certainly doing this analysis we should keep in mind the semiempirical nature of the CD calculations and semi-quantitative agreement to the experiment Several one-electron mixings and coupled oscillator interactions (nine in total) were analyzed in terms of changes of the interaction energies and the distances between the chromophores (Table 2). Three combinations of couplings between the near-UV CD transitions (*Lb-Lb*; *Lb-La* and *La-La*) of the same chromophore couple (W5 and W16) were analyzed. The average distance between the two tryptophans in the calculated MD snapshots is 6.8 Å, which is longer by 1.4 Å than the same distance in the crystal structure (Table 2). The averaged interaction energies (in absolute values) of the three combinations of coupling interactions (*Lb-Lb*, *Lb-La* and *La-La*) differ dramatically from that in the crystal structure, i.e. i) for the *Lb-Lb* coupling in the crystal structure the interaction energy is 0.32 cm^−1^ whilst in the MD snapshots the averaged value is 10.66 cm^−1^; ii) for the *Lb-La* coupling the values are 39.77 cm^−1^ (crystal structure) and 18.25 cm^−1^(averaged from the MD snapshots); and iii) for the *La-La* coupling the values are 67.04 cm^−1^ (crystal structure ) and 25.72 cm^−1^(MD averaged) (Table 2). In both sets of calculations the order of increasing interaction energies is as follows: *Lb-Lb* →*Lb-La* →*La-La*, however, in the MD-based calculations the differences between the values for the three couplings are much smaller to each other. The absolute values of the interaction energies in the three cases demonstrate a general trend of decreasing with the distance, however, this trend has specific form for each combination of transitions (Figure S4 in Supporting Information S1). As far as the distances and orientations are changed in the same manner, these differences would suggest that the structural fluctuations have slightly differential effect on each of the three combinations of transition interactions (between the same chromophores). The electric dipole moments of the Lb and La transitions have different orientations within the indole ring, and nevertheless the distance is the same, the angles between the transition moment vectors are different and lead to different interaction energies. Most importantly, the three types couplings within the same chromophore couple: Lb-Lb, Lb-La and La-La reflect to a different extend the fluctuation and relaxation of the external protein asymmetric field.

**Table 2:** Interactions between chromophores in terms of distances and net interaction energies in the crystal structures and as averaged from the MD trajectory in the wild type HCAII.

Interaction	Distance (Å)		Energy (cm^−1^)	
	Crystal Structure	MD	Crystal Structure	MD
*Lb*W5-*Lb*W16	5.4	6.8	0.32	10.66
*Lb*W5-*La*W16	5.4	6.8	39.77	18.25
*La*W5-*La*W16	5.4	6.8	67.04	25.72
*La*W97-*La*W245	8.0	7.7	7.80	6.72
*La*W192-*La*W209	10.4	11.1	12.27	10.27
*La*Y194-*La*W209	3.9	4.7	93.07	36.33
*La*Y88-*La*Y128	7.8	8.0	9.65	11.15
*Lb-La*W123	–	–	993.97	604.59
*Lb-La*W209	–	–	460.83	175.43

There are two clusters of distances between W5 and W16, found in the calculated snapshots: 4–5 Å and 7–10 Å, which demonstrate that the crystal structure alone (with distance 5.4 Å) is not fully representative for the geometric parameters of this coupled oscillator interaction under physiological conditions.

The coupling between *La* transitions of W97 and W245, as well as that between *La* transitions of W192 and W209, are characterized with considerably smaller differences in terms of both energies and distances between the crystal structure and the averaged structures from MD (Table 2). This behavior might reflect the fact that the orientations between the both chromophores are also not significantly changed. The tryptophan-tyrosine coupling between W209 and Y194 shows a considerable change in the interaction energy from 93.07 cm^−1^ (in the crystal structure) to 36.33 cm^−1^(in the MD-based structures), whilst the corresponding distances are changed less, i.e. 3.9 Å in the crystal structure and 4.7 Å in the averaged MD, which might suggest for larger changes in the orientations between the chromophores. The coupling between two tyrosines Y88 and Y128 shows closer values for both energies and distances in both set of structures. The pattern by which the interaction energy changes as a function of the distances for the above coupling interactions is very specific for each interaction as it can be seen in Figure S4 in Supporting Information S1. The conformational changes influence both the geometry (distance and orientation) and interaction energy of the coupling interactions in a distinct way for the individual chromophore couples.

The impact of the conformational fluctuations on the two strongest one electron mixings in the crystal structure was explored, namely the mixing between the *Lb* and *La* transitions of W123 and between the *Lb* and *La* transitions of W209 (Table 2 in the main text and Figure S5 in Supporting Information S1. The interaction energies here are again at least an order of magnitude higher than the ones for any of the coupled oscillator interactions. The averaged interaction energy of the *Lb-La* coupling of W123 (604.59 cm^−1^) from the MD snapshots differs considerably from that calculated for the crystal structure (993.97 cm^−1^) and the same applies for the *Lb-La* mixing within W209, which has values of 175.43 cm^−1^ (averaged from MD snapshots) and 460.83 cm^−1^(crystal structure). This suggests that the conformational changes influence not only the couplings between different chromophores but also exercise a strong effect on the mixing of transitions within the same chromophore by alternating the asymmetric field in which they are embedded. The results presented above are in agreement with studies on TEM-1 β-lactamase, which also indicate that structural changes influences both the nature of the mechanisms and the values of the rotational strengths in this enzyme ^30,31^. Recently the benefit of implementation of MD together with TDDFT was demonstrated in the case of CD spectra of tryptophan containing cyclic model dipeptides [37].

Subtle structural changes resulting from interactions with ligands, inhibitors and mutations could be monitored by experimental near-UC CD which reflects the aromatic interactions^2,10^. However, the CD mechanisms, generated by specific intra- and inter-chromophore interactions and therefore the contributions of the involved individual aromatic chromophores would differ in their response and sensitivity to conformational changes of similar magnitude (e.g. could be more or less sensitive). Therefore calculations might help in providing important complementary insight to experimental CD studies explaining the individual capability of aromatic chromophores to sense structural flexibility and relaxation.

The present analysis together with the achieved better agreement for the substantive near-UV spectral feature, calculated over the MD snapshots to the experimental one, shows the advantage of implementation MD simulations together with the crystal structures for an improved prediction of the protein CD spectra and achieving a deeper insight into their underlying mechanisms.

### CD Spectra of Tryptophan Mutants of HCAII Using the Crystal Structure and MD Simulations

**Figure 3:**
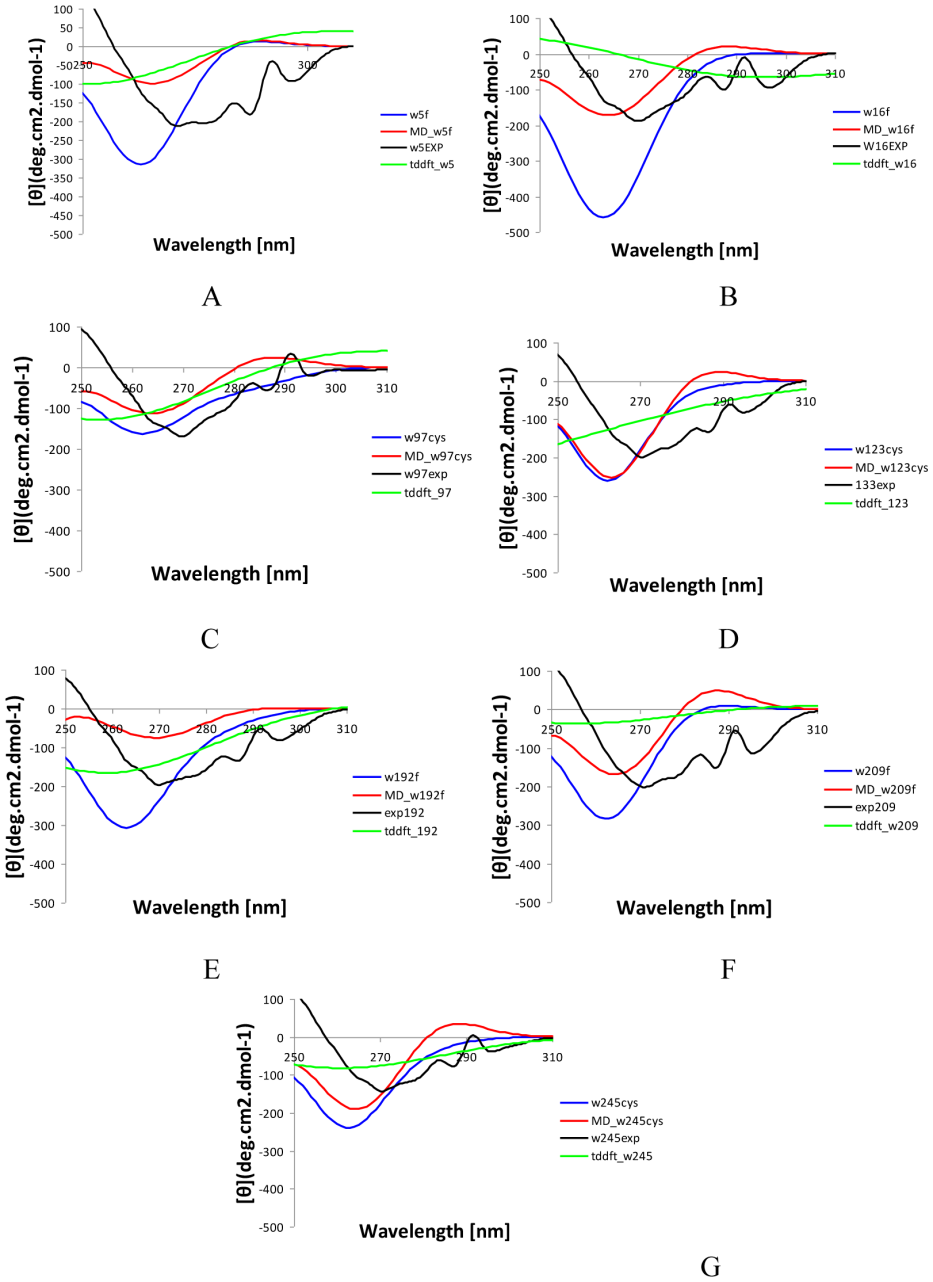
Calculated and experimental near-UV CD spectra of all tryptophan mutants of HCAII. The experimental spectra are shown *in black*, the predicted spectra with the matrix method based on single structure are shown *in blue*; the predicted spectra with the matrix method based on the MD snapshots are shown *in red*; TDDFT calculations are shown *in green*.

The CD spectra of the tryptophan mutants have been investigated experimentally and it was demonstrated that all of the tryptophan chromophores contribute over the entire UV CD region, with W97 and W245 providing the most significant contributions [8]. The calculations with the matrix method for all mutants, based on the *in silico* mutated and minimized crystal structures (Figure 3A–G, in blue), provided spectra in qualitative agreement with the experimental ones, with correctly predicted sign and relative magnitudes in the near- UV. The position of the minima is blue-shifted by approximately 7 nm in all mutants, as in the wild-type spectrum, which most likely reflects the nature of the theoretical model and parameters used.

The tryptophan mutations might lead to alterations in the local environment which cannot be effectively accounted for using a simple energy minimization technique. Instead, MD simulations were carried out before the excited states calculations. The calculations based on multiple snapshots randomly taken from the MD simulations of the mutants (Figure 3A–G, in red) in three cases, namely W16F, W209F and W245C, demonstrated a better agreement to the experimental spectra than in the single structure calculations and in two other cases, W97C and W192F, a slightly better agreement regarding the magnitudes was seen using the single structure. In the other two cases, W5F and W123C, the predictions based on both structural types were performed with similar success. It is important therefore that the computations of the CD spectra are performed cautiously, taking into account both the crystal structure and the snapshots from MD simulations.

The individual contributions of each tryptophan residue received as differential spectra between the wild type and each mutant were explored experimentally [8] and by computations using a single protein structure [9], [10]. We also calculated the individual tryptophans differential spectra using the both type of calculations - single structure and MD-averaged ones (Figure S6 in Supporting Information S1). MD-based calculations provided better agreement to the experimental differential CD spectra in cases of W5F W16F and W97C. The CD calculations based on a single structure presented better agreement to the experimental differential spectra for W123C, W192F, W245C and slightly better for W209F. It is important to note that there is not correlation between the performance of the single structure and MD-based calculations for the individual tryptophan mutants spectra and the corresponding differential ones. In the differential (individual tryptophan) spectra, possible accumulation of errors could take place because they are calculated as difference between the spectrum of the wild type enzyme and each mutant forms which leads to this difference. It is important therefore to reassert that CD calculations should be performed incorporating both the crystal structure and MD snapshots in strong correlation to the experimental CD spectra.

### Evaluating Restricted Structural Model Containing Only All Tryptophan and Tyrosine Chromophores Using the Matrix Method and TDDFT

**Figure 4:**
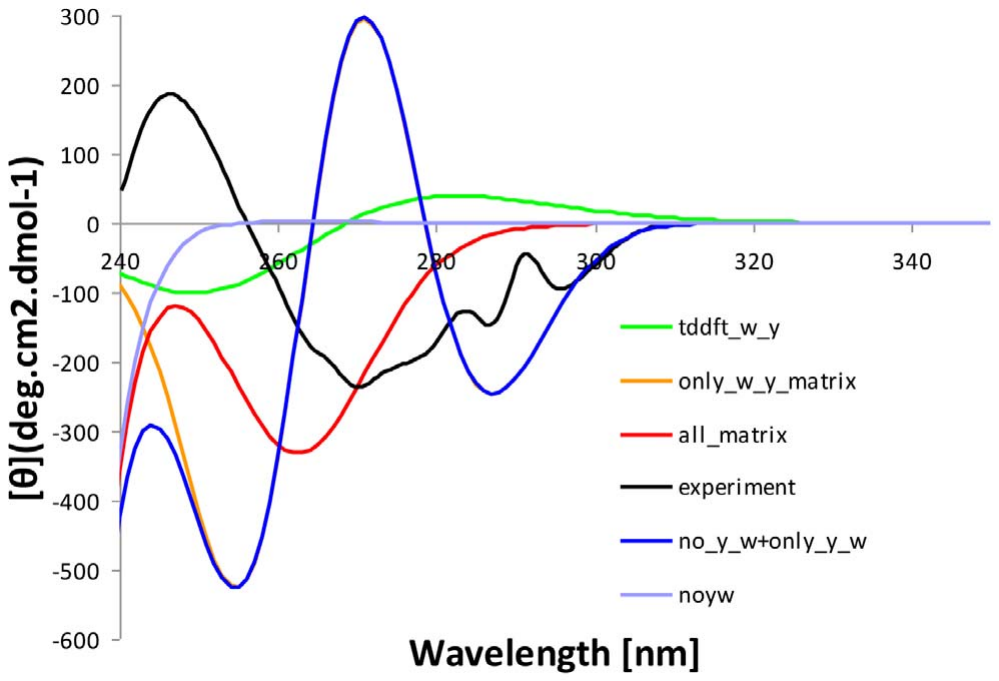
Comparison between the spectra calculated using Restricted Structural Model containing only the tryptophan and tyrosine chromophores (using TDDFT and the matrix method) and those calculated using the entire protein (using the matrix method).

Over the last several years TDDFT [16], [38] has became increasingly applied for calculating excited state properties of small and medium-sized molecules, many of which are of biological importance [39]. In order to evaluate the applicability of TDDFT calculations for larger multi-chromophore systems (such as HCAII), we computed the spectra of the wild-type enzyme, and all the seven tryptophan mutants, using B3LYP/6-31G(d) level of theory on a cluster of all tryptophan and tyrosine chromophores (kept at their positions from the crystal structure) in continuum solvent model environment with a dielectric constant of 4.0. Performing TDDFT calculation the entire protein structure (as in the case with the matrix method) is not feasible at present. Whilst the calculations were sensitive and distinguished between the wild-type enzyme and each mutant form, they did not reproduce the important spectral features (such as positions and magnitudes of the minima and maxima), even qualitatively (Figures 4 and 3A–G, in green).

Nevertheless, that the choice of the density functional and basis set could be extensively discussed (as for many recent excited state calculations e.g. [26], [37], [39]) and could contribute for the poor agreement between the calculated and the experimental spectra, more crucially the results might suggest that to calculate the CD properties at reasonable quality it is vitally important to include explicitly the protein environment. In order to test this hypothesis we carried out the matrix method of CD calculations on the tryptophans and tyrosines only (the same system which was used for TDDFT calculations). The resulting spectrum (Figure 4, in pink) is different from the TDDFT spectrum (in green) and has a deeper minimum, but is still too far from the experimental one. In addition the additive spectrum (Figure 4, in blue) from i) the spectrum calculated with only tryptophans and tyrosines by means of the matrix method (Figure 4, in pink) and ii) the spectrum calculated including all other chromophores without the aromatic ones by the same method (in yellow), does not provide the net spectrum (the one calculated using all chromophores including the aromatic ones with the matrix method) (in red). The result therefore confirms that the net CD spectrum is not a simple sum of the aromatic chromophores plus the rest of the protein but rather it is a complex function of multiple interactions between the aromatic chromophores incorporating the effect of the protein asymmetric field within a flexible environment. The study emphasizes the importance of explicit representation of the chromophore environment in agreement to other theoretical studies [40], [41].

Behind the demonstrated improvement of the spectra there are still effects, not included here, which might contribute to a better agreement between the experiments and the computations, such as polarization effects by the solvent, using larger number of snapshots and implementing better monopoles as parameters.

The results presented here confirm that, as in the case of TEM-1 β-lactamase [20], [32], [33], [42]–[46], the one electron effect (intra-chromophore transitions mixing) is the mechanism by which tryptophans generate the strongest CD contributions, whilst still keeping their ability to participate in coupling interactions (inter-chromophore mixing) with other chromophores (both tryptophans and tyrosines). Most importantly, the analysis provides comprehensive evidences for the substantial influence of the protein conformational flexibility on both mechanisms and intensities of the CD spectra.

### Conclusions

In this paper, we demonstrated that applying multilevel simulations in reference to multiple experimental data we could attain the synergy effect in understanding structure-spectra relationships. This approach not only provide opportunities in achieving a better agreement between the experimental and the calculated spectra, but provides enriched and deeper mechanistic insight in comparison to the single structure calculations. It reveals that the interactions between the aromatic chromophores (inter- and intra-chromophore ones) in proteins have a flexible and dynamic nature and drawing conclusions about them based solely on the crystal structure would not be representative. In order better agreement to the experimental CD spectra to be achieved the calculations should implement both the crystal structure and snapshots from MD trajectory. The results suggest that restricted structural model at both levels of theory: semiempirical one and TDDFT (B3LYP/6-31G(d) level) at present would not be a sustainable approach to reach improved accuracy for CD of proteins. Instead, better structural representation (e.g. multiple structures from MD), better parameters (monopoles) would be more efficient way to achieve such an improvement. The results reveal the crucial contribution of the protein environment (and its dynamics) for generating the CD properties and the vital importance of its explicit representation in the calculations (in contrast to the including only the aromatic chromospheres).

## Supporting Information

Supporting Information S1 Figure S1. TDDFT calculations of the HCAII wild-type CD spectrum with three different basis sets. Figure S2. RMSD values (in nm) along the 20 ns MD trajectory: A. The wild-type HCAII; B. The W5F mutant; C. The W16F mutant; D. The W97C mutant; E. The W123C mutant; F. The W192F mutant; G. The W209F mutant; and H. The W245C mutant. Figure S3. Calculations of the near-UV CD spectrum of HCAII using the matrix method: with all chromophores (*in green*); without tryptophans (*in blue*) and without tyrosines (*in red*). Figure S4. Distance dependence of the interaction energy for coupling interactions: A. between Lb-Lb, Lb-La and La-La transitions of W5 and W16; B. between W97 and W245; C. between W192 and W 209; D. between Y191 and W 209; E. between Y88 and Y125. Figure S5. Dependence of the interaction energy for the one electron (intra-chromophore) couplings between Lb and La transitions of W121 and between Lb and La transitions of W209 as a function of the snapshot. Figure S6. Differential near UV CD spectra of all tryptophan mutants of HCAII calculated as deference between the spectrum of the wild type and each of the tryptophan mutants. The experimental spectra are shown *in black*, the predicted spectra with the matrix method based on single structure are shown *in blue*; the predicted spectra with the matrix method based on the MD snapshots are shown *in red*;
(DOC)
