# Supplementary material for: Conformational Effects on the Circular Dichroism of Human Carbonic Anhydrase II: A Multilevel Computational Study
Source: PLoS One. 2013 Feb 25;8(2):e56874. doi: 10.1371/journal.pone.0056874 (PMC3582176; doi:10.1371/journal.pone.0056874)
Supplement: Supporting Information S1 — Figure S1. TDDFT calculations of the HCAII wild-type CD spectrum with three different basis sets. Figure S2. RMSD values (in nm) along the 20 ns MD trajectory: A. The wild-type HCAII; B. The W5F mutant; C. The W16F mutant; D. The W97C mutant; E. The W123C mutant; F. The W192F mutant; G. The W209F mutant; and H. The W245C mutant. Figure S3. Calculations of the near-UV CD spectrum of HCAII using the matrix method: with all chromophores (in green); without tryptophans (in blue) and without tyrosines (in red). Figure S4. Distance dependence of the interaction energy for coupling interactions: A. between Lb-Lb, Lb-La and La-La transitions of W5 and W16; B. between W97 and W245; C. between W192 and W 209; D. between Y191 and W 209; E. between Y88 and Y125. Figure S5. Dependence of the interaction energy for the one electron (intra-chromophore) couplings between Lb and La transitions of W121 and between Lb and La transitions of W209 as a function of the snapshot. Figure S6. Differential near UV CD spectra of all tryptophan mutants of HCAII calculated as deference between the spectrum of the wild type and each of the tryptophan mutants. The experimental spectra are shown in black, the predicted spectra with the matrix method based on single structure are shown in blue; the predicted spectra with the matrix method based on the MD snapshots are shown in red; (DOC) [file pone.0056874.s001.doc]

**SUPPORTING INFORMATION**

Figure S1. TDDFT calculations of the HCAII wild-type CD spectrum with three different basis sets.

A B

C D

E F

G

Figure S2. RMSD values (in nm) along the 20 ns MD trajectory: A. The wild-type HCAII; B. The W5F mutant; C. The W16F mutant; D. The W97C mutant; E. The W123C mutant; F. The W192F mutant; G . The W209F mutant; and H. The W245C mutant.

Figure S3. Calculations of the near-UV CD spectrum of HCAII using the matrix method: with all chromophores (*in green*); without tryptophans (*in blue*) and without tyrosines (*in red*).

A B

C D

E

Figure S4. Distance dependence of the interaction energy for coupling interactions: A. between Lb-Lb, Lb-La and La-La transitions of W5 and W16; B. between W97 and W245; C. between W192 and W 209; D. between Y191 and W 209; E. between Y88 and Y125.

Figure S5. Dependence of the interaction energy for the one electron (intra-chromophore) couplings between Lb and La transitions of W121 and between Lb and La transitions of W209 as a function of the snapshot.

A

B

C

D

E

F

G

Figure S6: Figure caption. Differential near UV CD spectra of all tryptophan mutants of HCAII calculated as deference between the spectrum of the wild type and each of the tryptophan mutants. The experimental spectra are shown *in* *black*, the predicted spectra with the matrix method based on single structure are shown *in blue*; the predicted spectra with the matrix method based on the MD snapshots are shown *in* *red*;
